# Bovine Colostrum: Human and Animal Health Benefits or Route Transmission of Antibiotic Resistance—One Health Perspective

**DOI:** 10.3390/antibiotics12071156

**Published:** 2023-07-06

**Authors:** Carla Miranda, Gilberto Igrejas, Patrícia Poeta

**Affiliations:** 1 Microbiology and Antibiotic Resistance Team (MicroART), Department of Veterinary Sciences, University of Trás-os-Montes e Alto Douro, 5000-801 Vila Real, Portugal; carlisabelmi@utad.pt; 2 Toxicology Research Unit (TOXRUN), Advanced Polytechnic and University Cooperative (IUCS-CESPU), University Institute of Health Sciences, 4585-116 Gandra, Portugal; 3 Associated Laboratory for Green Chemistry (LAQV-REQUIMTE), University NOVA of Lisbon, 1099-085 Caparica, Portugal; gigrejas@utad.pt; 4 Department of Genetics and Biotechnology, University of Trás-os-Montes e Alto Douro, 5000-801 Vila Real, Portugal; 5 Functional Genomics and Proteomics Unit, University of Trás-os-Montes e Alto Douro, 5000-801 Vila Real, Portugal; 6 Veterinary and Animal Research Centre (CECAV), University of Trás-os-Montes e Alto Douro, 5000-801 Vila Real, Portugal; 7 Associate Laboratory for Animal and Veterinary Science (AL4AnimalS), University of Trás-os-Montes e Alto Douro, 5000-801 Vila Real, Portugal

**Keywords:** bovine colostrum, antibiotic residues, antimicrobial resistance, bioactive compounds, health benefits

## Abstract

After calving, bovine colostrum is obtained from the mammary gland of the dam in the first days and fed to newborn ruminant to prevent microbial infections. Each bovine colostrum has a unique biochemical composition with high nutraceutical value compared to milk. However, bovine colostrum is influenced by various factors, such as environmental, individual, and genetic factors, as well as processing methods. Proper colostrum management is crucial for obtaining high-quality colostrum and mitigating bacterial contamination. This is important not only for the health and survival of calves but also for the health of humans who consume colostrum and its co-products. It is essential to ensure that the consumed colostrum is free of pathogens to reap its benefits. Health-promoting products based on colostrum have gained significant interest. However, colostrum can contain pathogens that, if not eliminated, can contribute to their transmission and spread, as well as antibiotic resistance. The aim of this review was to promote the animal and human health benefits of bovine colostrum by improving its microbial quality and highlighting potential routes of dissemination of antibiotic-resistant pathogens. Implementing hygienic measures is one of the key factors in mitigating colostrum bacterial contamination and obtaining safe and high-quality colostrum. This helps reduce the exposure of pathogens to newborn calves, other animals, and humans, in a One Health analysis.

## 1. Introduction

Bovine colostrum is considered the first postpartum secretion or milk obtained from the mammary gland of dairy cows up to the third day [1,2,3]. The same authors also consider colostrum as the first secretion from the first eight milkings or up to five days post-partum, including the transition milk [2,3,4].

Colostrum possesses high nutraceutical value, including a variety of nutrients and/or biologically active compounds, such as immunoglobulins, proteins, nucleotides, vitamins, growth factors, carbohydrates, and enzymes. Although its physicochemical composition is highly variable and depends on factors, such as nutrition, age, genetics and the environment, these compounds are vital in promoting immunological defense, nutrition, as well as the growth and development of newborns [1,2,3,4,5]. Additionally, colostrum plays a key role in the endocrine system, metabolism, nutritional state, gut function, and colonization.

In particular, the high level of immunoglobulins present in the colostrum confers resistance against several pathogenic agents, providing passive immunity to the neonate, through gut absorption, as the gut is highly permeable during the first 24 h of life [2,6,7]. Since neonates are born agammaglobulinemic (with an absence of blood immunoglobulins), the ingestion of cow colostrum is the only way to obtain immunity and is crucial for the survival and immediate and long-time health of the calves. Ideally, colostrum ingestion of at least 4 L should occur within the first 4 h after calving [5,8,9,10,11]. In the United States, only 39% of collected bovine colostrum met the requirements for microbiological parameters and immunoglobulin levels (>50 g/L of IgG), while 60% showed poor quality, increasing the risk of passive immunity failure and/or bacterial infections in calves [12]. Failure of passive immunity is associated with lower performance of animals, weakened immune system, reduced animal production profitability, and consequently, increased susceptibility to disease, morbidity, and mortality, leading to significant losses in production, breeding, and economics [13]. Furthermore, male calves receive poorer quality and smaller quantities of colostrum over a longer period of time after birth [14]. However, there are specific situations where newborn calves are not fed with maternal colostrum, such as in cases of multiple births, cows with acute mastitis or infection with *Mycoplasma bovis* and *Mycobacterium avium* subsp. *paratuberculosis*, or maladapted behavior common in first lactation heifers [15].

The management practices and preservation methods of colostrum are determining factors in maintaining high-quality colostrum, as well as its microbial quality [10]. In case of excess colostrum, it can be preserved and later administrated when poor production is observed or for other purposes, using chemical preservatives, heat-treatment (pasteurization), low temperature (freezing and refrigeration) [2,10,15], or other described methods like fermentation with *Lactobacillus*, ultrasonication and dehydration [16]. The ideal methods should not interfere with the colostrum’s chemical composition and should permit the mitigation of microbial contamination. Several bacterial pathogens have been identified into colostrum that will be administrated to calf neonates like *Listeria monocytogenes*, which harbors several virulence-associated genes [17], *Enterococcus* spp. [18], enterobacteria, *Escherichia coli,* and aerobic mesophilic spore-forming bacteria [16]. Although many studies report milk pathogens, studies reporting colostrum microbial contamination are limited. Consequently, calves fed with colostrum containing residues of antibiotics can increase the risk of developing antibiotic resistance and contribute to its dissemination to humans through the food chain, other animals, and the environment via eliminated feces [19].

The investigation of the potential use and valorization of bovine colostrum and/or its derivatives has increased. Current studies have shown very promising results in animal and human health with the application of bovine colostrum and its derivatives, mainly for the prevention of gastrointestinal tract diseases and respiratory tract infections. Bovine colostrum has been used in several functional foods, feed, pharmaceuticals, and nutraceuticals for both animals and humans [3,4,5]. Furthermore, pathogen-specific antibodies and polyclonal antibodies obtained from colostrum, also known as hyperimmune bovine colostrum, are used as vaccines against bovine and human pathogens, neutralizing rotavirus, human immunodeficiency virus, *E. coli*, *Streptococcus mutans*, *Shigella, Vibrio cholerae*, *Clostridioides difficile*, *Candida albicans*, *Cryptosporidium* spp. and *Helicobacter pylori* [20,21].

Since studies on the clinical benefits of bovine colostrum are limited and presented poor methodological quality and lack replication, further studies are needed for the safe clinical application of colostrum [22]. The aim of this review was to promote the animal and human health benefits of bovine colostrum and highlight possible routes of dissemination of antibiotic-resistant pathogens. The search for relevant articles was performed on PubMed and ScienceDirect databases between March and May 2023, using keywords such as ‘bovine colostrum’, ‘bovine colostrum’ and ‘antimicrobial resistance’, and ‘bovine colostrum’ and ‘health benefits’.

## 2. Importance of Bioactive Components and Animal Health Benefits

During the last three weeks of a cow’s pregnancy, colostrum is produced in the mammary gland. Bovine colostrum obtained during the first milking includes fat, protein such as immunoglobulins, casein and albumin, nonspecific antimicrobial factors, growth factors, leukocytes, hormones, vitamins, and nutrients [10]. Some of these bioactive components exhibit immune, inflammatory, biological and bacteriostatic properties (Table 1) [13]. Each bovine colostrum has a unique biochemical composition, although it is influenced by environmental factors (season of calving), individual factors (age and nutrition of dam), and genetic factors (breed), as well as processing methods [4,10].

The high nurturing and biological value of colostrum changes quickly, with these properties reducing to levels similar to milk within each hour after calving (after 120 h). Therefore, the earlier colostrum is fed to calves, especially within the first hours of life, the more beneficial it will be for them, mainly due to the intestinal absorption capacity of immunoglobulins [13]. Without the development of its own active immunity system, the calf depends on the passive transfer of immunoglobulins by colostrum, particularly IgG and IgM, for its immunity and health. Maternal leukocytes transferred through colostrum also activate the cell-mediated immune response [10]. Similar results were obtained by Arfuso et al. [23], showing a positive correlation of immune response, including leukocyte populations like lymphocytes, neutrophils and monocytes, between cows and calves around calving. During the first day of life of the newborn calves, the presence of pro-inflammatory cytokines like interleukin 6 is positively influenced by the interleukin 6 levels in cows, which are also transferred by colostrum [23]. The white blood cells and monocytes count, as well as interleukin 6 concentration, increase in calves up to 15 days of age, demonstrating that these parameters are influenced by their age, except the neutrophil levels. On the contrary, cortisol levels, which are associated with stress and immune-modulatory markers, decrease after 24 h from colostrum intake [23].

Colostrum contains a high concentration of protein, nutrients and growth factors that are essential for muscle development and energy. Fat and lactose (the most representative carbohydrate) contribute to body temperature regulation and thermogenesis in calves, serving as the main source of energy. Lactose also plays an important role in stimulating the functions of nerve cells (brain and spinal cord) and the development of organs like the liver, heart, and kidneys [13,24]. The presence of lysozyme, lactoperoxidase and lactoferrin in colostrum performs germicidal and bacteriostatic functions [13].

Vitamins, which are more concentrated into colostrum than milk, such as vitamin A, carotene, vitamin E, riboflavin, folic acid, vitamin B12, choline, and selenium, play various important roles, including defense mechanisms, immunity, stabilization of the cell membranes, antioxidation and digestion/absorption of lipids [10,13,24].

Minerals, which are also found at higher levels into colostrum compared to milk, such as magnesium, calcium, phosphorus, zinc and sodium chloride, except for potassium levels [10,13,24]. Zinc, iron and copper levels in colostrum decrease after 336 h post-calving [9]. Deficiency in these mineral components can lead to problems in the development and health of calves, such as muscular dystrophies, poor growth, diarrhea, infections, and death [13].

**Figure 1:**
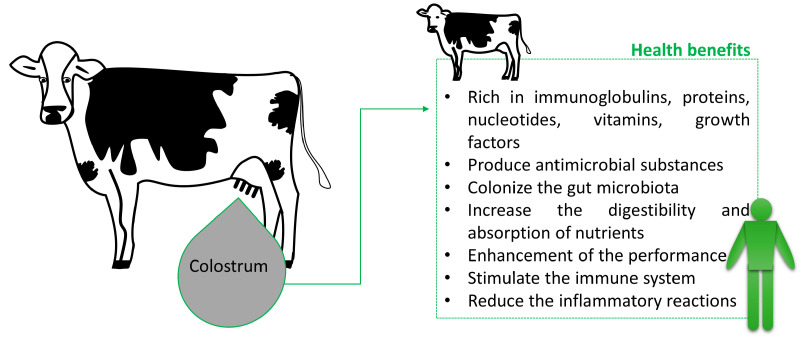
Animal and human health benefits of bovine colostrum.

Some studies have demonstrated the use of colostrum as a feed additive in other animal species like animal production and pets [5]—during the first days of life when they have immature gut and immune functions [25]. In poultry production, supplementation with bovine colostrum has shown improvements in protein quality, feed conversion efficiency, and growth performance, reducing feed costs and mortality rates [4,26]. In general, when it is consumed as a food supplement, bovine colostrum enhances the immune functions (Figure 1); gut microbiota; and the performance of animals like dogs, cats, horse, and fish [5,27,28,29].

## 3. Human Health Benefits

Bovine colostrum and/or its derivatives have been consumed by humans for a long time. Potential applications of bovine colostrum have been investigated for human health and nutrition [30]. Nowadays, research on colostrum supplementation in neonates and adult humans has increased due to its antibacterial properties, resulting in more than 130 clinical trials conducted [5], although more studies are required to confirm the clinical outcomes observed in low number of tests performed *in vitro*, animals, and/or humans.

The development of colostrum as an ingredient and its separation in fractions to increase food bioactivity and potential health benefits is promising but also a challenge, according to Arslan et al. [30]. The high level of protein content is another advantage of using colostrum. Additionally, several immune effects mediated by lactoferrin, casein and immunoglobulins, as well as growth factors have been investigated in humans [30]. Bovine colostrum acts on immune cells, such as lymphocytes, cytokines, macrophages, natural killer and dendritic cells [31].

Bovine colostrum is used in infants due to prebiotic properties, increasing enteral protein intake and the intestinal adhesion of *Lactobacillus* and *Bifidobacterium*. Preterm infants fed with bovine colostrum in combination with human milk reduce the feed intolerance, severity of neonatal sepsis, and mortality but do not influence the incidence of necrotizing enterocolitis and length of hospital stay [32]. However, oropharyngeal administration of colostrum applied to preterm infants with a gestational age ≤ 32 weeks reduces the incidence of necrotizing enterocolitis, sepsis and length of hospital stay [8].

In athletes during intensified training, bovine colostrum supplementation has demonstrated beneficial effects, ameliorating the performance, gut permeability, and immune functions, and reducing the infection risk of upper respiratory tract [33,34]. An experiment in rat models demonstrated that the application of colostrum in combination with honey on cutaneous lesions mitigated scars and exudates, accelerating the growth of granulation tissue [35].

Like several animal and human trials, the ingestion of bovine colostrum, particularly in infants and children, has shown health benefits as immunomodulatory nutritional and antimicrobial supplement, being effective and safe. It reduces or prevents infections, diarrhea, growth-failure, short-bowel syndrome, preterm birth, mucositis, and necrotizing enterocolitis [5,25]. However, it is not recommended to completely substitute human milk with bovine colostrum due to nutritional imbalances between them. Intolerance and allergies have not been reported in association with consuming bovine colostrum [25]. Although the bioactive factors and immunoglobulins increase their action, these should be preserved during all processing and thermal treatment to obtain safe bovine colostrum with high nutritional quality [25].

## 4. Bovine Colostrum Pathogens

Bovine colostrum constitutes the first potential exposure of calves to pathogenic agents. Colostrum may contain pathogens (Table 2) that can be transmitted to calves, affecting animal health and colostrum quality [36,37]. Additionally, the intestinal absorption of immunoglobulins is reduced by high bacteria levels in colostrum. Despite the implementation of several strategies and measures to reduce bacterial contamination, colostrum frequently exhibits high levels of coliform bacteria. Pathogens, such as *Staphylococcus* spp., *Streptococcus uberis*, coliforms and gram-negative rods have been identified in colostrum [36].

Good practices in colostrum management (harvest, storage and feeding) include cleaning and sanitizing the udders and bucket before milking. Clean and disinfected equipment is essential for colostrum transfer to reduce bacterial contamination. Until colostrum administration to calves, it should be stored at low temperatures (refrigerated or frozen) or subjected to pasteurization at high temperatures. Pasteurization is recommended at 60 °C to maintain the IgG level in colostrum and permit the elimination of pathogens like *Salmonella* Enteritidis, *E. coli,* and *Mycoplasma bovis* [2,10,14,38].

The pathogens identified into colostrum can have different origins [36,39,40], including:

- Normal inhabitants of the mucosa and skin of dam (*Streptococcus* spp., *Staphylococcus*, *Pasteurella* spp., *Corynebacterium* spp. and *Arconobacterium pyogenes*);
- Environmental contaminants (gram-negative rods, *Bacillus* spp. and *Micrococcus* spp.);
- Fecal contaminants (coliforms, *Enterococcus* spp., *Streptococcus bovis*, *E. coli* and *Proteus* spp.);
- Mammary pathogens (*Streptococcus uberis*, *Staphylococcus aureus* and *Streptococcus dysgalactiae*);
- During the collection, processing, storage and feeding processes;
- Systemic infections (*Listeria* spp., *Salmonella* spp. and *Staphylococcus* spp.).

From infected cows or cows with mammary gland infections—such as *Salmonella* Enteritidis, *E. coli*, *Mycoplasma bovis*, *Mycobacterium avium* subsp. *paratuberculosis*, *Campylobacter jejuni*, *Streptococcus* spp., *Staphylococcus aureus*, and *Listeria monocytogenes*—these can be transmitted to calves through colostrum [17,39,41,42]. In Holstein Black cows, colostrum collected within the first 6 h after calving was detected as predominantly *Staphylococcus* spp., including *S. aureus*, but also *Listeria innocua*, *Enterococcus feacium*, *Enterococcus faecalis*, *Bacillus clausii* and *Macrococcus caseolyticus* [37].

In the United States, 39% of bovine colostrum comprised good microbial requirements as total plate count (<100,000 colony-forming unit (CFU)/mL) and total coliform count (<10,000 CFU/mL). The remaining colostrum samples revealed a risk of bacterial infections to calves [12]. Similarly, analyzed colostrum from Czech dairy farms also showed heavy microbial contamination for the following parameters: gram-negative non-coliform count (<5000 CFU/mL), total plate count, and total coliform count. Furthermore, the majority of bacterial pathogens isolated from colostrum samples were originated from environmental and fecal contaminants, and commensal microbiota of bovine mucosa and skin. However, potential pathogens, such as *Enterococcus* spp., *E. coli*, *Streptococcus parauberis*, *Streptococcus uberis*, *Staphylococcus aureus* and *Streptococcus dysgalactiae* were also found [40].

The consumption of bovine colostrum contaminated with pathogens can have a negative impact on calf health, increasing mortality rates. For humans, consuming raw colostrum without thermal treatment can also pose risks [37,39]. On the other hand, immunoglobulin absorption decreased under the presence of high levels of microbial contamination into colostrum [10,41,43,44]. The temperature of storing and the thermal treatments applied to colostrum affect bacterial levels. Cummins et al. [43] showed that warmer temperatures (13 °C or 22 °C) used during colostrum storage were more likely to increase the bacterial counts, reducing pH and IgG concentration relative to unpasteurized colostrum that are fed immediately after sampling, pasteurized colostrum, and fresh colostrum stored at 4 °C for 2 days.

## 5. Antibiotic Residues and Antibiotic-Resistant Bacteria in Colostrum

The transmission of bacterial pathogens through colostrum is a concern for animal health and food safety. In this regard, colostrum can also serve as a potential route for the colonization of calves’ gastrointestinal tract by antibiotic-resistance bacteria and their dissemination [18]. Several studies have reported the level of colostrum microbial contamination at various stages, from calving to ingestion [12,40]. However, studies that have specifically examined the presence of antibiotic-resistant bacteria and antimicrobial compounds in colostrum are limited.

In 2016, the European Food Safety Authority (EFSA) evaluated two main risks associated with calves developing antibiotic resistance due to the consumption of colostrum: (1) feeding calves with colostrum containing antibiotic residues and (2) feeding calves with colostrum/milk of cows that have been administrated antibiotics during the lactation and dry period [19].

Cephalosporins, penicillin and penicillin combined with aminoglycosides are the most frequently administrated antibiotics during these stages. Although milk may contain substantial residues during antibiotic treatment performed during lactation or the dry period, feeding calves with contaminated milk is more likely to disseminate antibiotic-resistant bacteria among the calves. The EFSA displays thermal inactivation as a measure to reduce the presence of these bacteria in colostrum or raw milk [19]. Colostrum from cows treated with antibiotics is used to feed calves because it cannot be sold, and its elimination is difficult. Additionally, colostrum contributes to resistance against infections and promotes calf growth [45,46].

The presence of antimicrobial residues in colostrum depends on the drug, formulation and nature, and the time between cow treatment and calving. Gonggrijp et al. [47] detected antimicrobial residues above the limit for cloxacillin (29%), ampicillin (3%), and penicillin (1%) in bovine colostrum. Other studies analyzed the presence of antimicrobial-resistant bacteria shed in feces from milk calves fed with antimicrobials. The results showed that cows treated with these antibiotics did not shed these bacteria in colostrum [48,49]. Langford et al. [48] showed that low antimicrobial concentration like penicillin in milk fed to calves, did not have an effect on the fecal shedding of penicillin-resistant bacteria, but higher antibiotic concentrations increased shedding.

### Presence of Antibiotic-Resistant Bacteria

Feeding milk with a low concentration of penicillin, ceftiofur, ampicillin, tetracycline hydrochloride, oxytetracycline and neomycin sulfate increases the number of antimicrobial-resistant *E. coli* shedding in calves [50,51]. Analyzing the feeding of calves with colostrum from cows treated with aminoglycosides and penicillin during the dry period, it was found that there was no increase in shedding of antimicrobial-resistant *E. coli* [47,52]. Another study observed a significant association between an increase in extended-spectrum beta-lactamases (ESBL)/AmpC-producing *E. coli* in the feces of calves and a low total coliform count in colostrum [47]. A current study showed that the majority of *Enterococcus* spp. isolated from bovine colostrum are multidrug resistant (≥3 antimicrobial classes resistance), indicating that colostrum contaminated with multidrug-resistant bacteria acts as a reservoir and a vehicle for their transmission [18]. In another study, ESBL-producing *E. coli* carrying *bla*_CTX-M-15_ and *bla*_TEM-171_ genes were detected in colostrum fed to dairy calves, and similar results were obtained from fecal samples. Additionally, these isolates showed high resistance to tetracycline, ciprofloxacin, ampicillin, and kanamycin [53]. Antimicrobial-resistant *E. coli* is more frequently identified in fecal samples of young calves compared to adult cattle, indicating that these young animals serve as reservoirs [54,55]. Oh et al. [54] demonstrated that antimicrobial resistant *E. coli* begin to be shed at 2 to 3 days old. The tetracyclines-associated resistant gene, *tet*(B), was identified in maternal colostrum and fecal samples. Most of the *E. coli* isolated from fecal samples were resistant to tetracycline, sulfisoxazole and streptomycin. Additionally, neonatal calves that have been treated with ceftiofur acquired and shed *E. coli* resistant to beta-lactams by 21–28 days of age, indicating an increased likelihood of antibiotic resistance. These findings suggest that the transmission of *E. coli* resistant to beta-lactams can occur through feeding colostrum [55,56]. In general, the implementation of policy changes on farms has demonstrated a trend towards decreased antimicrobial resistance and changes in resistance patterns. Farm managers have taken steps to mitigate antibiotic use [56]. Furthermore, low usage and low levels of antimicrobial resistance for highly important antimicrobials in human medicine have been observed [55,57].

**Figure 2:**
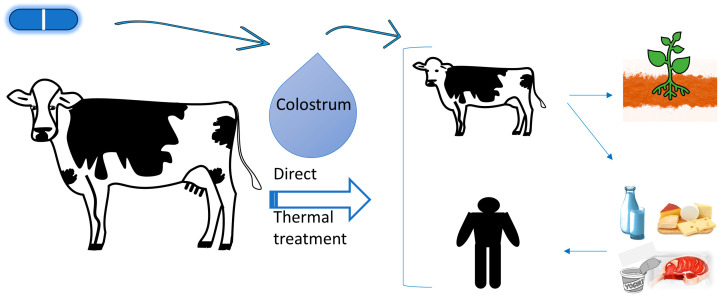
Potential niches contaminated with antibiotics or their residues and antimicrobial-resistant bacteria through the consumption of bovine colostrum.

The presence of antibiotic-resistant bacteria and antibiotic residues in fed colostrum contributes to the development of infections/diseases and the selection of antibiotic-resistant pathogens in calves. Moreover, the shedding of calves’ feces into the environment with antibiotic-resistant bacteria and antibiotic residues constitutes a direct and global risk to public health (Figure 2). The obtained milk from treated cows is also a direct risk to humans through dairy products and unpasteurized milk [19,58,59].

## 6. Measures to Mitigate Bacterial Contamination in Bovine Colostrum

The high-quality colostrum, including the microbial quality, is influenced by management practices and preservation methods [10]. Several measures have been implemented to maintain the colostrum free of bacterial pathogens, like chemical and thermal treatments, ultrasonication, and dehydration methods [2,10,15,16]. Therefore, colostrum from cows infected with known pathogens, such as *Mycobacterium avium* subsp. *paratuberculosis*, should not be used for feeding, and pooling of raw colostrum should be avoided [10]. The equipment and feeding process are the main critical control sources to prevent and minimize colostrum bacterial contamination [60]. Evaluation of the bacterial contamination of colostrum-feeding equipment like esophageal tube feeders, nipple bottles, and pails can be performed by conventional laboratory bacterial counts or by using rapid and effective on-farm alternatives like adenosine triphosphate bioluminescence swabs, such as AquaSnap Total and MicroSnap Coliform, as described by Renaud et al. [61]. This study reported that 59% of colostrum-feeding equipment exceeded the total bacterial count and 21% of them exceeded the total coliform count.

Hygienic and sanitary practices should start before milking, with the cleaning and sanitizing of udders and buckets. During colostrum harvesting, it should be transferred to clean and sanitized equipment to reduce bacterial contamination [10]. Bacteria existent in colostrum are more likely to multiply at room temperatures [62].

To preserve the microbial quality of colostrum until its administration to calves, colostrum should be stored at low temperatures (refrigerated or frozen) or at high temperatures by pasteurization, or through the addition of chemical preservatives and bacterial cultures [15]. Bovine colostrum should be refrigerated (4 °C) or frozen (−20 °C) for up to 1 h after harvest [10]. Although high-temperature treatment (>60 °C for 60 min) is effective to reduce the total bacterial count, Malik et al. [62] highlighted that low-temperature treatment (≤60 °C during 60 min) reduces the loss of colostrum IgG concentration and increases the serum IgG concentration and the serum total protein relative to high treatment, and also eliminates potential pathogens like *Mycoplasma bovis*, *Salmonella* Enteritidis, *E. coli*, *Clostridium* spp., and *Mycobacterium avium* subsp. *paratuberculosis*—which is promising for the colostrum industry [10,60,62]. Another study also demonstrated a decrease in microbial counts into colostrum while maintaining IgG concentrations when colostrum was subjected to pasteurization at 60 °C for 60 min [63]. In addition, low temperatures and pH were demonstrated to reduce bacterial growth [15,60]. Heat treatment of colostrum is a helpful measure that reduces overall bacteria counts, controlling pathogenic bacteria. However, it has a considerable associated cost, according to Denholm [15].

When potassium sorbate preservative was added into fresh colostrum during the 96 h of refrigeration period, it was demonstrated to be the most effective treatment in reducing the bacterial counts, such as total coliform counts and total plate count, compared to other treatments where colostrum was only refrigerated or stored at ambient temperature without or with potassium sorbate preservative [10,60]. However, no potassium sorbate is effective against *Mycoplasma* spp. Although there are other chemical preservatives with on-farm or laboratory use for acidified colostrum, such as citric acid, sodium benzoate or formic acid, their use required careful consideration as they may have advantages for specific groups of bacteria but may impair the IgG absorption levels, nutritional content, and raise safety concerns [15]. Regarding bacterial preservation of bovine colostrum, the addition of bacterial cultures (yoghurt cultures and *Lactobacillis*) and aerobic fermentation are not effective during storage. On the other hand, anaerobic fermentation has shown promising results as an alternative to the low temperature method in reducing pathogens, maintaining nutritional contents and IgG concentration and increasing storage time [15].

Other recent methods for colostrum preservation at room temperature, such as UV light radiation, lyophilization, and high-pressure processing have been in development, but studies are limited. These methods are only applicable in a laboratory setting and not on-farm and these may destroy immunoglobulin levels and be ineffective against most bacteria [15]. Another study showed that a combination of three treatments (ultrasonication, dehydration, and fermentation with *Lactobacillis*) reduced microbial contamination, but further research is needed [16].

## 7. Conclusions

Feeding calves with colostrum of good microbial quality provides optimal advantages for their health compared to milk. However, the presence of potential pathogens, including antibiotic-resistant bacteria, or antibiotic residues from cows treated with antibiotics has negative effects on their health and growth. Furthermore, animals fed with contaminated colostrum can contribute to environmental dissemination and the food chain. Considering these factors, measures of sanitary and hygiene, as well as thermal treatment of colostrum should be considered to mitigate this silent pandemic of antimicrobial resistance and maximize the benefits of colostrum usage in animals and humans.

## Figures and Tables

**Table 1:** Main bioactive components identified in bovine colostrum.

Bioactive Component	Functions
Fat	Body temperature regulate and energy
Lactose	Body temperature regulate and energy, stimulate of the nervous system
Protein (Immunoglobulins)	Immune response
Other protein (casein and albumin)	Muscle development and energy
Nonspecific antimicrobial factors	Immune response
Growth factors	Muscle development and energy
Leucocyte populations	Immune response
Hormones	Growth performance
Vitamins	Immune response and antioxidant
Nutrients	Muscle development and energy
Cytokines (Interleukin 6)	Immune and anti-inflammatory response
Enzymes (lysozyme, lactoferrin and lactoperoxidase)	Antimicrobial response
Minerals	Growth performance

**Table 2:** Main potential bacterial pathogens identified in bovine colostrum.

Bacterial Pathogens	
Gram-Positive	Gram-Negative
*Staphylococcus aureus*	*Escherichia coli*
*Streptococcus uberis*	*Salmonella* Enteritidis
*Streptococcus parauberis*	*Campylobacter jejuni*
*Streptococcus dysgalactiae*	*Mycoplasma bovis*
*Enterococcus faecalis*	
*Enterococcus feacium*	
*Listeria innocua*	
*Listeria monocytogenes*	
*Mycobacterium avium* subsp. *paratuberculosis*	

## Data Availability

All the data are contained within the article.

## References

[1] Zarcula S., Cernescu H., Mircu C., Tulcan C., Morvay A., Baul S., Popovici D. (2010). Influence of breed, parity and food intake on chemical composition of first colostrum in cow. Anim. Sci. Biotechnol..

[2] McGrath B.A., Fox P.F., McSweeney P.L.H., Kelly A.L. (2016). Composition and properties of bovine colostrum: A review. Dairy Sci. Technol..

[3] Poonia A., Shiva (2022). Bioactive compounds, nutritional profile and health benefits of colostrum: A review. Food Prod. Process. Nutr..

[4] Mehra R., Garhwal R., Sangwan K., Guiné R.P.F., Lemos E.T., Buttar H.S., Visen P.K.S., Kumar N., Bhardwaj A., Kumar H. (2022). Insights into the Research Trends on Bovine Colostrum: Beneficial Health Perspectives with Special Reference to Manufacturing of Functional Foods and Feed Supplements. Nutrients.

[5] Linehan K., Ross R.P., Stanton C. (2023). Bovine Colostrum for Veterinary and Human Health Applications: A Critical Review. Annu. Rev. Food Sci. Technol..

[6] Tsioulpas A., Grandison A.S., Lewis M.J. (2007). Changes in physical properties of bovine milk from the colostrum period to early lactation. J. Dairy Sci..

[7] Stelwagen K., Carpenter E., Haigh B., Hodgkinson A., Wheeler T.T. (2009). Immune components of bovine colostrum and milk. J. Anim. Sci..

[8] OuYang X., Yang C.Y., Xiu W.L., Hu Y.H., Mei S.S., Lin Q. (2021). Oropharyngeal administration of colostrum for preventing necrotizing enterocolitis and late-onset sepsis in preterm infants with gestational age ≤ 32 weeks: A pilot single-center randomized controlled trial. Int. Breastfeed. J..

[9] Abd El-Fattah A.M., Abd Rabo F.H., El-Dieb S.M., El-Kashef H.A. (2012). Changes in composition of colostrum of Egyptian buffaloes and Holstein cows. BMC Vet. Res..

[10] Godden S.M., Lombard J.E., Woolums A.R. (2019). Colostrum Management for Dairy Calves. Vet. Clin. N. Am. Food Anim. Pract..

[11] Lopez A.J., Heinrichs A.J. (2022). Invited review: The importance of colostrum in the newborn dairy calf. J. Dairy Sci..

[12] Morrill K.M., Conrad E., Lago A., Campbell J., Quigley J., Tyler H. (2012). Nationwide evaluation of quality and composition of colostrum on dairy farms in the United States. J. Dairy Sci..

[13] Puppel K., Gołębiewski M., Grodkowski G., Slósarz J., Kunowska-Slósarz M., Solarczyk P., Łukasiewicz M., Balcerak M., Przysucha T. (2019). Composition and Factors Affecting Quality of Bovine Colostrum: A Review. Animals.

[14] Renaud D.L., Steele M.A., Genore R., Roche S.M., Winder C.B. (2020). Passive immunity and colostrum management practices on Ontario dairy farms and auction facilities: A cross-sectional study. J. Dairy Sci..

[15] Denholm K. (2022). A review of bovine colostrum preservation techniques. J. Dairy Res..

[16] Bartkiene E., Bartkevics V., Ikkere L.E., Pugajeva I., Zavistanaviciute P., Lele V., Ruzauskas M., Bernatoniene J., Jakstas V., Klupsaite D. (2018). The effects of ultrasonication, fermentation with *Lactobacillus* sp., and dehydration on the chemical composition and microbial contamination of bovine colostrum. J. Dairy Sci..

[17] Hasegawa M., Iwabuchi E., Yamamoto S., Esaki H., Kobayashi K., Ito M., Hirai K. (2013). Prevalence and characteristics of *Listeria monocytogenes* in bovine colostrum in Japan. J. Food Prot..

[18] Cunha S., Soares R., Maia M., Igrejas G., Silva F., Miranda C., Poeta P. Presence of antibiotic-resistant *Enterococcus faecalis* in colostrum supplied to calves?. Proceedings of the 1st International Electronic Conference on Antibiotics—The Equal Power of Antibiotics and Antimicrobial Resistance.

[19] Ricci A., Allende A., Bolton D., Chemaly M., Davies R., Fernández Escámez P.S., Girones R., Koutsoumanis K., Lindqvist R., EFSA Panel on Biological Hazards (BIOHAZ) (2017). Risk for the development of Antimicrobial Resistance (AMR) due to feeding of calves with milk containing residues of antibiotics. EFSA J..

[20] Ulfman L.H., Leusen J.H.W., Savelkoul H.F.J., Warner J.O., Van Neerven R.J. (2018). Effects of bovine immunoglobulins on immune function, allergy, and infection. Front. Nutr..

[21] Borad S.G., Singh A.K., McSweeney P.L.H., McNamara J.P. (2022). Immunoglobulins. Encyclopedia of Dairy Sciences.

[22] Rathe M., Müller K., Sangild P.T., Husby S. (2014). Clinical applications of bovine colostrum therapy: A systematic review. Nutr. Rev..

[23] Quigley J. (2002). Passive immunity in newborn calves. Adv. Dairy Technol..

[24] Arfuso F., Minuti A., Liotta L., Giannetto C., Trevisi E., Piccione G., Lopreiato V. (2023). Stress and inflammatory response of cows and their calves during peripartum and early neonatal period. Theriogenology.

[25] Sangild P.T., Vonderohe C., Melendez Hebib V., Burrin D.G. (2021). Potential Benefits of Bovine Colostrum in Pediatric Nutrition and Health. Nutrients.

[26] Afzal I., Khan A.A., Khaliq T., Hamadani H., Shafi M., Raja T.A. (2017). Effect of bovine colostrum supplemented diets on performance of broiler chicken. Indian J. Poult. Sci..

[27] Giffard C.J., Seino M.M., Markwell P.J., Bektash R.M. (2004). Benefits of bovine colostrum on fecal quality in recently weaned puppies. J. Nutr..

[28] Gore A.M., Satyaraj E., Labuda J., Engler R., Sun P., Kerr W., Conboy-Schmidt L. (2021). Supplementation of diets with bovine colostrum influences immune and gut function in kittens. Front. Vet. Sci..

[29] Moretti D.B., Nordi W.M., Machado-Neto R. (2019). Redox balance and tissue development of juvenile Piaractus mesopotamicus subjected to high stocking density and fed dry diets containing nutraceutical food. Lat. Am. J. Aquat. Res..

[30] Arslan A., Kaplan M., Duman H., Bayraktar A., Ertürk M., Henrick B.M., Frese S.A., Karav S. (2021). Bovine Colostrum and Its Potential for Human Health and Nutrition. Front. Nutr..

[31] Ghosh S., Iacucci M. (2021). Diverse Immune Effects of Bovine Colostrum and Benefits in Human Health and Disease. Nutrients.

[32] Ismail R.I.H., Awad H.A., Imam S.S., Gad G.I., Aboushady N.M., Abdou R.M., Eissa D.S., Azzam N.T., Barakat M.M., Yassin M.M. (2021). Gut priming with bovine colostrum and T regulatory cells in preterm neonates: A randomized controlled trial. Pediatr. Res..

[33] Jones A.W., March D.S., Thatcher R., Diment B., Walsh N.P., Davison G. (2019). The effects of bovine colostrum supplementation on in vivo immunity following prolonged exercise: A randomised controlled trial. Eur. J. Nutr..

[34] March D.S., Marchbank T., Playford R.J., Jones A.W., Thatcher R., Davison G. (2017). Intestinal fatty acid-binding protein and gut permeability responses to exercise. Eur. J. Appl. Physiol..

[35] Tanideh N., Abdordideh E., Yousefabad S.L.A., Daneshi S., Hosseinabadi O.K., Samani S.M. (2017). Evaluation of the healing effect of honey and colostrum in treatment of cutaneous wound in rat. Comp. Clin. Pathol..

[36] Fecteau G., Baillargeon P., Higgins R., Paré J., Fortin M. (2002). Bacterial contamination of colostrum fed to newborn calves in Québec dairy herds. Can. Vet. J..

[37] Baltrukova S., Zagorska J., Eihvalde I. (2019). Preliminary study of bovine colostrum quality in Latvia. Res. Rural Dev..

[38] Poulsen K.P., Foley A.L., Collins M.T., McGuirk S.M. (2010). Comparison of passive transfer of immunity in neonatal dairy calves fed colostrum or bovine serum-based colostrum replacement and colostrum supplement products. J. Am. Vet. Med. Assoc..

[39] Alegbeleye O.O., Guimarães J.T., Cruz A.G., Sant’Ana A.S. (2018). Hazards of a ‘Healthy’ Trend? An Appraisal of the Risks of Raw Milk Consumption and the Potential of Novel Treatment Technologies to Serve as Alternatives to Pasteurization. Trends Food Sci. Technol..

[40] Šlosárková S., Pechová A., Staněk S., Fleischer P., Zouharová M., Nejedlá E. (2021). Microbial contamination of harvested colostrum on Czech dairy farms. J. Dairy Sci..

[41] Elizondo-Salazar J.A., Heinrichs A.J. (2008). Review: Heat treating bovine colostrum. Prof. Anim. Sci..

[42] Lima S.F., Teixeira A.G.V., Lima F.S., Ganda E.K., Higgins C.H., Oikonomou G., Bicalho R.C. (2017). The bovine colostrum microbiome and its association with clinical mastitis. J. Dairy Sci..

[43] Cummins C., Berry D.P., Murphy J.P., Lorenz I., Kennedy E. (2017). The effect of colostrum storage conditions on dairy heifer calf serum immunoglobulin G concentration and preweaning health and growth rate. J. Dairy Sci..

[44] Abuelo A., Havrlant P., Wood N., Hernandez-Jover M. (2019). An investigation of dairy calf management practices, colostrum quality, failure of transfer of passive immunity, and occurrence of enteropathogens among Australian dairy farms. J. Dairy Sci..

[45] Godden S. (2008). Colostrum management for dairy calves. Vet. Clin. N. Am. Food Anim. Pract..

[46] Brunton L.A., Duncan D., Coldham N.G., Snow L.C., Jones J.R. (2012). A survey of antimicrobial usage on dairy farms and waste milk feeding practices in England and Wales. Vet. Rec..

[47] Gonggrijp M., Scherpenzeel C., Kappert C., Heuvelink A., Holtstege M., Nijenhuis E., Tijs S., Keurentjes J., Lam T., Velthuis A. (2015). Resistentieontwikkeling bij Jonge Kalveren.

[48] Aust V., Knappstein K., Kunz H.J., Kaspar H., Wallmann J., Kaske M. (2013). Feeding untreated and pasteurized waste milk and bulk milk to calves: Effects on calf performance, health status and antibiotic resistance of faecal bacteria. J. Anim. Physiol. Anim. Nutr..

[49] Langford F.M., Weary D.M., Fisher L. (2003). Antibiotic resistance in gut bacteria from dairy calves: A dose response to the level of antibiotics fed in milk. J. Dairy Sci..

[50] Pereira R.V., Siler J.D., Ng J.C., Davis M.A., Warnick L.D. (2014). Effect of preweaned dairy calf housing system on antimicrobial resistance in commensal *Escherichia coli*. J. Dairy Sci..

[51] Berge A.C., Moore D.A., Sischo W.M. (2006). Field trial evaluating the influence of prophylactic and therapeutic antimicrobial administration on antimicrobial resistance of fecal *Escherichia coli* in dairy calves. Appl. Environ. Microbiol..

[52] Duse A., Waller K.P., Emanuelson U., Unnerstad H.E., Persson Y., Bengtsson B. (2015). Risk factors for antimicrobial resistance in fecal *Escherichia coli* from preweaned dairy calves. J. Dairy Sci..

[53] He Z., Yang S., Ma Y., Zhang S., Cao Z. (2021). Detection of CTX-M-15 Extended-Spectrum β-Lactamases Producing *Escherichia coli* Isolates from Colostrum and Faeces of Newborn Dairy Calves in China. Pathogens.

[54] Oh S.I., Ha S., Roh J.H., Hur T.Y., Yoo J.G. (2020). Dynamic Changes in Antimicrobial Resistance in Fecal *Escherichia coli* from Neonatal Dairy Calves: An Individual Follow-Up Study. Animals.

[55] Jarrige N., Cazeau G., Bosquet G., Bastien J., Benoit F., Gay E. (2020). Effects of antimicrobial exposure on the antimicrobial resistance of *Escherichia coli* in the digestive flora of dairy calves. Prev. Vet. Med..

[56] Afema J.A., Davis M.A., Sischo W.M. (2019). Antimicrobial use policy change in pre-weaned dairy calves and its impact on antimicrobial resistance in commensal *Escherichia coli*: A cross sectional and ecological study. BMC Microbiol..

[57] Uyama T., Renaud D.L., Morrison E.I., McClure J.T., LeBlanc S.J., Winder C.B., de Jong E., McCubbin K.D., Barkema H.W., Dufour S. (2022). Associations of calf management practices with antimicrobial use in Canadian dairy calves. J. Dairy Sci..

[58] Chee-Sanford J.C., Mackie R.I., Koike S., Krapac I.G., Lin Y.F., Yannarell A.C., Maxwell S., Aminov R.I. (2009). Fate and transport of antibiotic residues and antibiotic resistance genes following land application of manure waste. J. Environ. Qual..

[59] Firth C.L.L., Kremer K., Werner T., Käsbohrer A. (2021). The Effects of Feeding Waste Milk Containing Antimicrobial Residues on Dairy Calf Health. Pathogens.

[60] Stewart S., Godden S., Bey R., Rapnicki P., Fetrow J., Farnsworth R., Scanlon M., Arnold Y., Clow L., Mueller K. (2005). Preventing bacterial contamination and proliferation during the harvest, storage, and feeding of fresh bovine colostrum. J. Dairy Sci..

[61] Renaud D.L., Kelton D.F., LeBlanc S.J., Haley D.B., Jalbert A.B., Duffield T.F. (2017). Validation of commercial luminometry swabs for total bacteria and coliform counts in colostrum-feeding equipment. J. Dairy Sci..

[62] Malik M.I., Rashid M.A., Raboisson D. (2022). Heat treatment of colostrum at 60 °C decreases colostrum immunoglobulins but increases serum immunoglobulins and serum total protein: A meta-analysis. J. Dairy Sci..

[63] Donahue M., Godden S.M., Bey R., Wells S., Oakes J.M., Sreevatsan S., Stabel J., Fetrow J. (2012). Heat treatment of colostrum on commercial dairy farms decreases colostrum microbial counts while maintaining colostrum immunoglobulin G concentrations. J. Dairy Sci..

